# Influence of zein-coated magnesium oxide nanoparticle reinforcement on the flexural strength of polymeric denture base resins

**DOI:** 10.3389/fdmed.2026.1895756

**Published:** 2026-09-21

**Authors:** Afaf A. Almabadi, Ghada H. Naguib, Mohamed T. Hamed, Jumana Mazhar, Othman S. Alsulaimani, Lulwa Al-Turki

**Affiliations:** 1 Department of Oral and Maxillofacial Prosthodontics, Faculty of Dentistry, King Abdulaziz University, Jeddah, Saudi Arabia; 2 Department of Restorative Dentistry, Faculty of Dentistry, King Abdulaziz University, Jeddah, Saudi Arabia; 3 Department of Oral Biology, Cairo University School of Dentistry, Cairo, Egypt; 4 Department of Fixed Prosthodontics, Cairo University School of Dentistry, Cairo, Egypt; 5 King Abdulaziz University, Jeddah, Saudi Arabia; 6 Harvard Medical School, Harvard University, Boston, MA, United States

**Keywords:** anti-bacterial agents, dentures, flexural strength, nanoparticles, polymethyl methacrylate

## Abstract

**Background/objectives:**

Conventional polymethyl methacrylate denture base resins exhibit limitations in mechanical durability and microbial resistance. Zein-coated magnesium oxide nanoparticles (zMgO NPs) have previously demonstrated antimicrobial activity when incorporated into dental polymers. However, their influence on mechanical performance has not been thoroughly characterized. This study investigated whether integrating zMgO NPs affects the flexural strength of heat-polymerized and autopolymerizing acrylic denture base materials.

**Methods:**

Five commercially available polymethyl methacrylate resins (three heat-cured and two self-cured) were evaluated. Sixty samples of each type were randomly assigned to four groups based on nanoparticle concentration: 0%, 0.3%, 0.5%, and 1% by weight. Sample fabrication and testing were performed in accordance with ISO 20795-1 for denture base polymers. Flexural strength was assessed using a three-point bending test. Data were analyzed using two-way analysis of variance followed by *post hoc* comparisons with statistical significance set at 0.05.

**Results:**

Samples containing 0.5% and 1% zMgO NPs demonstrated significantly higher flexural strength than controls, whereas the 0.3% zMgO NPs groups showed no statistically significant difference compared to the control.

**Conclusions:**

Incorporation of zMgO NPs maintains the flexural performance of denture base polymers. Higher nanoparticle loadings produced significantly higher flexural strength, suggesting potential for multifunctional denture materials with improved structural durability. Future work should include long-term aging and simulated oral conditions to confirm clinical applicability.

## Introduction

Edentulism remains a prevalent condition worldwide, particularly among older adults, and is associated with functional, nutritional, and psychosocial consequences (1). Removable dentures continue to be a common treatment option for restoring oral function in edentulous patients, particularly when circumstances don't allow for a fixed prosthesis (2). Among available denture base polymers, polymethyl methacrylate (PMMA) is the most frequently selected material in clinical prosthodontics due to its balance of handling characteristics, affordability, and biological compatibility (3). It is the key component in both heat-cured and self-cured acrylic resins, which are used in denture base manufacturing and denture repair (4, 5). It has remained the dominant material because it combines clinical compatibility with practical manufacturability and economic accessibility (6, 7).

Despite its widespread use, PMMA prostheses remain susceptible to structural and biological limitations. These include brittleness, fatigue-related degradation, dimensional changes during polymerization, and surface properties that may facilitate microbial accumulation. Such weaknesses contribute to fracture risk, particularly along stress-prone regions such as the midline, where cyclic loading and material fatigue can lead to failure over time (8, 9). Fractures can develop from functional stresses, user habits, denture handling, resin porosity, thin areas within denture bases, and crack formation (10, 11). John et al. reported that 68% of acrylic denture base failures happen within 3 years of manufacturing, mainly due to deformation from masticatory loads, leading to fatigue fractures (12). Another major limitation is the reduced mechanical durability of repaired denture bases, which contributes to a shorter interval to re-fracture incidents and increased patient visits and costs (13–15).

Microbial colonization of denture surfaces is another clinically relevant concern. Biofilm accumulation beneath prostheses has been strongly associated with denture stomatitis, an inflammatory condition affecting mucosal tissues. This condition is common among denture wearers and is influenced by hygiene practices, prosthesis fit, systemic health status, and medication use. Candida species, especially *Candida albicans*, are frequently implicated in its development (16). The condition is very common, with studies showing a prevalence of 15%–80% among denture wearers worldwide (17–19).

Efforts to improve denture base performance have increasingly focused on nanoscale reinforcement approaches, as nanomaterials can alter polymer behavior through interfacial interactions that influence stress transfer, chain mobility, and surface characteristics. However, their effectiveness depends strongly on particle composition, concentration, and dispersion within the host material (13, 14, 20–25). Azmy et al. previously investigated the mechanical performance of modifying PMMA acrylic resin with 3% and 7% zirconium dioxide, silicon dioxide, and titanium dioxide nanoparticles. Their study concluded that zirconium dioxide improved PMMA's flexural and impact strength at both concentrations, while silicon dioxide, and titanium dioxide only showed improvement at low concentrations (22).

Ideally, the addition of the nanoparticles should improve or maintain the inherent esthetics, biocompatibility, mechanical strength, and dimensional stability of the acrylic resin (26). The lack of a standardized consensus on evaluation protocols and biological implications complicates the selection of nanoparticles. It is therefore crucial that the selected nano-fillers demonstrate not only functional and mechanical benefits but also biological safety and biocompatibility.

Magnesium oxide nanoparticles (MgO NPs) have recently been popularized as a reinforcement for different dental materials (27, 28). However, their performance depends on achieving stable dispersion, since particle clustering can reduce functional surface area and limit reinforcement efficiency (29). Surface modification strategies have therefore been developed to enhance stability within polymer matrices (30). This is achieved through zein, a natural protein-based polymer that constitutes approximately 50%–70% of the endosperm protein in corn (31). It is frequently used for various textile, food, and pharmaceutical indications (32). Its use as a carrier is cost-effective and allows for efficient utilization of by-products (33). When incorporated with nanoparticles, this polymer can be used as a coating, limiting hydrophobic interactions and stabilizing the surface tension that arises from a lack of atoms available to bond to the nanoparticles (32, 33). Zein has successfully been used with MgO NPs to provide antimicrobial benefits (34, 35).

Previous investigations have demonstrated that zein-coated magnesium oxide nanoparticles (zMgO NPs) confer strong antimicrobial effects when incorporated into dental materials (36–39). The significance of adding an antimicrobial effect to acrylic resin denture bases is especially important, as a denture resistant to *Candida albicans* may help lower the occurrence of denture stomatitis (16).

Although the biological benefits of this nanoparticle system have been reported, its influence on the mechanical performance of PMMA denture base polymers has not been fully characterized. In particular, limited information exists regarding how varying concentrations of zMgO NPs affect flexural strength, a critical property governing clinical longevity. The present study aimed to address this gap by evaluating the impact of adding zMgO NPs at concentrations of 0.0%, 0.3%, 0.5%, and 1.0% on the flexural strength of various types of PMMA-based denture base resins. The null hypothesis was that there were no significant differences in flexural strength among the different types of acrylic resin at all nanoparticle concentrations.

## Methodology

### Materials

Five commercially available PMMA denture base materials were evaluated, including three heat-polymerized resins (ProBase Hot, Ivoclar Vivadent, Schaan, Liechtenstein; Vertex Heat-Cured, Vertex Dental, Soesterberg, The Netherlands; DPI Heat-Cure, Dental Products of India, Fort, Mumbai) and two autopolymerizing formulations (Vertex Self-Cured, Vertex Dental, Soesterberg, The Netherlands; GC Nature-Cryl Pour, GC International AG, Lucerne, Switzerland). The zein polymer was obtained from Sigma–Aldrich (St. Louis, USA). MgO nanoparticles were synthesized in-house. Zein coating of the MgO NPs was performed in this study. Other chemical agents were obtained from local vendors.

This study employed a balanced 5 × 4 factorial design with 5 acrylic resin types and 4 nanoparticle concentrations (0%, 0.3%, 0.5%, and 1%), yielding 20 experimental groups. Using G*Power (Version 3.1.9.6), assuming an effect size of *f* = 0.25, a significance level of 0.05, and 80% power, a minimum total sample size of 289 samples was required (40). To ensure adequate power and maintain equal group sizes, 300 samples were included, corresponding to 15 samples in each of the 20 experimental groups (Figure 1). Samples were first prepared according to the assigned acrylic resin type. Within each acrylic resin type, samples were randomly assigned to one of four zMgO NP concentration groups (0%, 0.3%, 0.5%, or 1%) using a computer-generated random number sequence to ensure unbiased allocation to the nanoparticle concentration groups.

**Figure 1:**
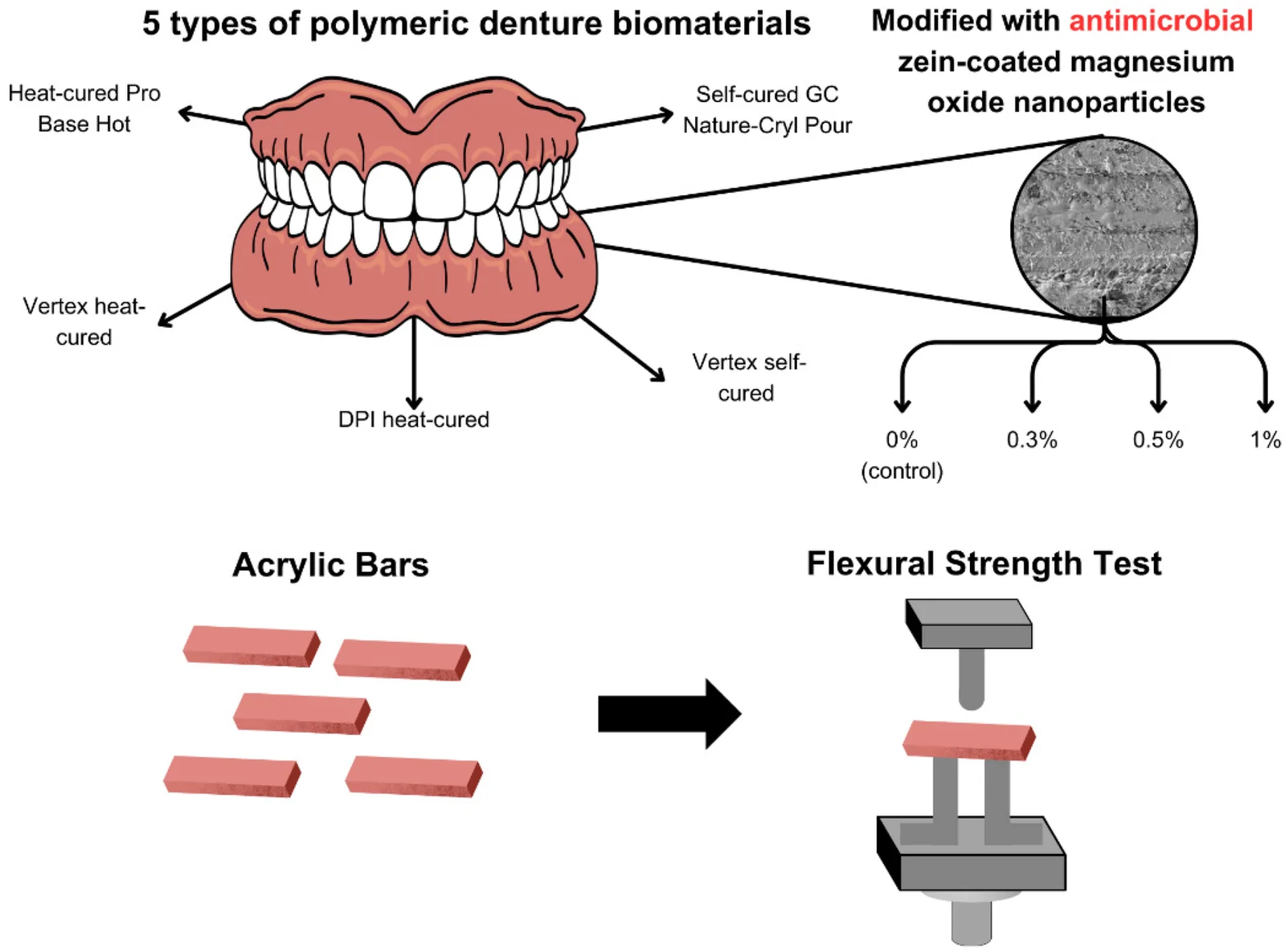
An overview of the distribution of the acrylic types (three heat-polymerized types: proBase Hot, Vertex heat-cured, DPI heat-cured; two autopolymerizing types: Vertex self-cured and GC nature-cryl pour) and nanoparticle concentrations (0%, 0.3%, 0.5%, and 1%). The bars were subjected to a 3-point bending test to assess flexural strength.

### Preparation of nanoparticles

Portions of the nanoparticle synthesis procedure were adapted from our previously published work (34, 35). MgO NPs were produced through a controlled precipitation and thermal conversion approach. The magnesium acetate precursor solution (6.44 g with 75 mL of distilled water, dissolved for 30 min at 25 °C) was combined with urea (1.2 g with 25 mL of distilled water), subjected to autoclaving (15 min), and subsequently purified through centrifugation and filtration steps. The resulting product was dried (60 °C for 24 h) and calcined (600 °C for 1 h) to obtain crystalline MgO particles.

Surface stabilization was achieved using the zein coating (32–34, 41). The polymer (0.02 g) was dissolved in alkaline ethanol and added dropwise to a polyvinyl alcohol dispersion (15 mL of 0.9% w/v) containing MgO (0.02 g) at 10 °C, with ultrasonic shear applied to promote uniform coating (20 kHz, 750 W). The suspension was stirred to allow solvent evaporation (500 rpm, room temp), purified by centrifugation (300 rpm, 45 min), suspended in buffer (5 mL), and lyophilized following the addition of a cryoprotectant (2% w/v trehalose).

### Sample preparation

Polymerization of both heat-cured and self-cured materials was performed according to the manufacturer's specified protocols. ProBase Hot polymerizes by preparing the mixture at a 2.25:1 polymer-to-monomer ratio, placing the closed flask in cold water, heating it up to 100 °C for 45 min, and allowing it to cool at room temperature for 30 min. Vertex Heat-Cured uses a 2.2:1 polymer-to-monomer ratio and polymerizes by placing the preparation in cold water, heating it to 100 °C for 30 min, and air-cooling to ambient temperature. DPI Heat-Cure is prepared by mixing the powder and liquid at a 3:1 ratio, packing the dough into the flask, and curing in a water bath at either 74 °C for 2 h or 100 °C for 30 min. Vertex Self-Cured uses a polymer-to-monomer ratio of 1.7:1 and polymerizes after mixing in using a pressure vessel at 55 °C at 2½ bar for 10 min. GC Nature-Cryl Pour requires a polymer-to-monomer ratio of 1.43:1 and polymerizes in a pressure vessel 20 PSI for 30 min.

For each acrylic resin, the polymer-to-monomer ratio was maintained according to the manufacturer's recommendations. The required mass of zMgO nanoparticles was calculated as a percentage of the polymer powder weight (0%, 0.3%, 0.5%, or 1%) and added before mixing with the monomer. Weight calibration was accurate to 0.0001 grams (Mettler Toledo, Fischer Scientific, USA) (Table 1). Mixing was performed with mechanical vortex agitation to ensure homogeneous distribution (2,000 rpm). Sample fabrication, dimensions, conditioning, storage, and flexural strength testing were performed in accordance with ISO 20795-1 for denture base polymers. Following finishing and polishing, all samples were conditioned by immersion in distilled water at 37 °C for 48 h, in accordance with ISO 20795-1, before mechanical testing.

**Table 1:** Material composition for each experimental group.

Material	Nanoparticle concentration	MgO NPs weight (g)	Powder weight (g)	Monomer (mL)
PROBASE	Control	0	225	100
	0.3%	0.6	225	100
	0.5%	1.1	225	100
	1%	2.2	225	100
DPI HC	Control	0	300	100
	0.3%	0.9	300	100
	0.5%	1.5	300	100
	1%	3	300	100
VERTEX HC	Control	0	220	100
	0.3%	0.6	220	100
	0.5%	1.1	220	100
	1%	2.2	220	100
GC SC	Control	0	143	100
	0.3%	0.4	143	100
	0.5%	0.7	143	100
	1%	1.4	143	100
VERTEX SC	Control	0	170	100
	0.3%	0.5	170	100
	0.5%	0.8	170	100
	1%	1.7	170	100

Bar-shaped samples measuring 65 mm in length × 10 mm in width × 3 mm in thickness were fabricated for each group (42–44). Heat-polymerized samples were prepared using conventional flasking procedures with wax patterns formed in silicone molds. The wax samples were invested in the dental flask and immersed in hot water to eliminate the wax. After dewaxing, the acrylic resin was filled into the mold space and pressed using a hydraulic press. Then, it was cured according to the manufacturer's instructions, after which the samples were de-flasked and wet-polished using silicon carbide papers. The autopolymerizing samples were formed in silicone molds and polymerized under pressure. Finished samples were retrieved and similarly refined by wet-polishing with silicon carbide abrasive papers.

### Flexural strength test

Prior to mechanical testing, the samples were coded with the allocated group number by an independent investigator. The operator performing the mechanical tests was blinded to the sample group allocation (acrylic resin type and zMgO NP concentration) throughout data collection.

To assess flexural strength in accordance with ISO 20795-1, a 3-point bending test was conducted on a universal testing machine (Instron 5944, Instron 5000 Premier Testing Systems series, Instron® Mechanical Testing Systems). The samples were loaded onto the machine and tested at a crosshead speed of 0.5 mm/min using a 50 kN loading cell, with the support span length set to 50 mm (45). Flexural strength was calculated using the following Formula 1:

$$S=\;\frac{3FL}{2bd^{2}}$$ (1)

*S* = the flexural strength (MPa); *F* = the maximum load applied to the acrylic resin samples (Newton); *L* = the span in between the supports; *b* and *d* are, respectively, the width and height of the acrylic resin samples in mm.

### Statistical analysis

Statistical analysis was performed using SPSS software (Version 23, IBM Corp., Armonk, USA). Two-way ANOVA was used to evaluate the effects of acrylic resin type and nanoparticle concentration on flexural strength. When significant differences were identified, *post hoc* pairwise comparisons were performed using Tukey's test. The level of statistical significance was set at 0.05.

## Results

Two-way ANOVA demonstrated a statistically significant effect of acrylic resin type on flexural strength (*p* *<* 0.05; *p* = 0.025). Across all materials, a consistent concentration-dependent increase in flexural strength was observed with increasing zMgO NPs content (Figure 2 and Table 2). No statistically significant differences were identified between the control samples and those containing 0.3% zMgO NPs for any of the tested materials (*p* > 0.05 for all groups; ProBase HC: *p* = 0.32; DPI HC: *p* = 0.07; Vertex HC: *p* = 0.36; GC SC: *p* = 0.77; Vertex SC: *p* = 0.22).

**Figure 2:**
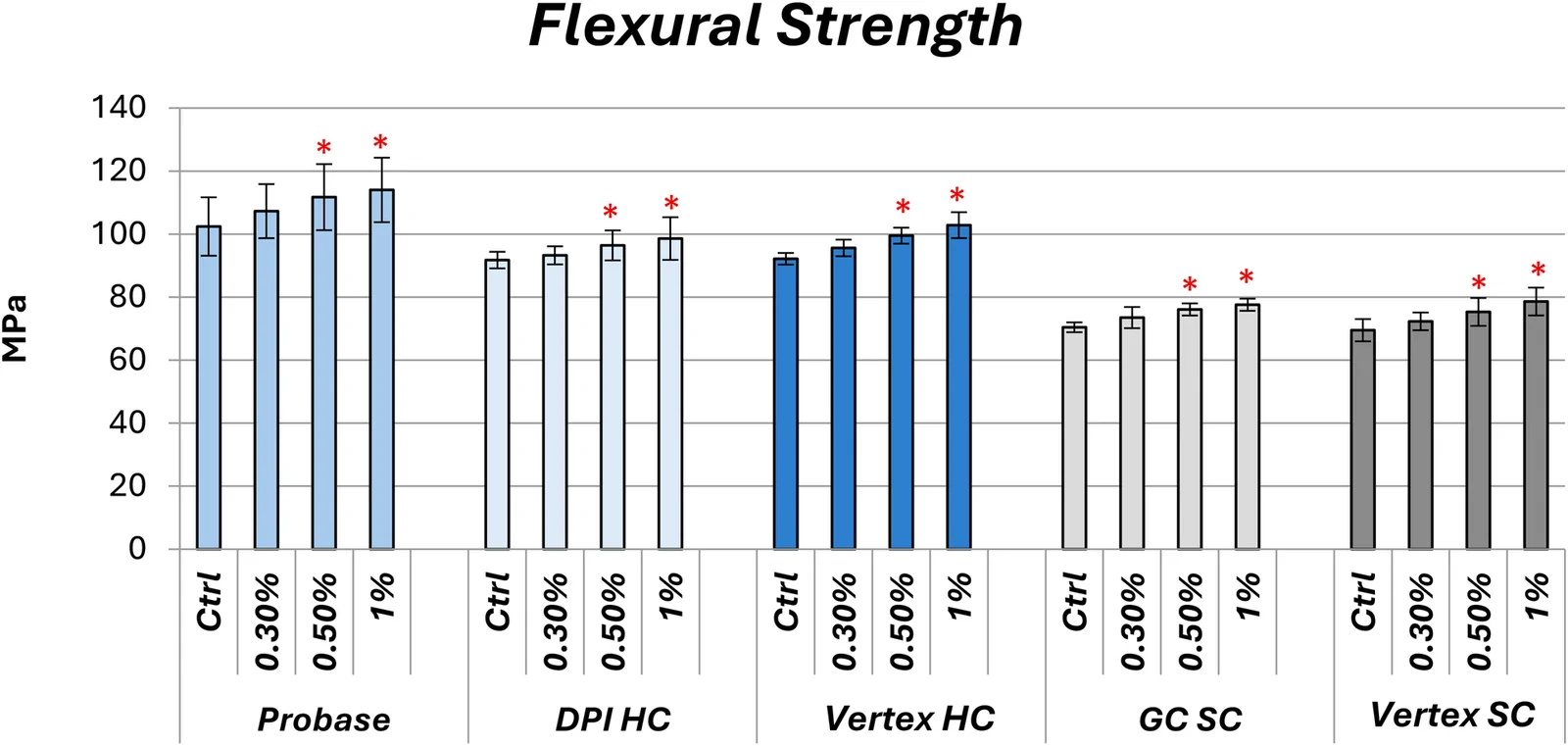
Comparison of the flexural strengths found with the different acrylic resins at various concentrations of zMgO NPs. All groups at 0.3% concentration of zMgO NPs had no significant difference when compared to the control (*p* > 0.05), whereas all groups at 0.5% and 1% zMgO NPs had statistically significant differences when compared to the control (*p < 0.05). Probase: ProBase Hot (heat-cured), DPI HC: DPI Heat-Cure, Vertex HC: Vertex Heat-Cured, GC SC: GC Nature-Cryl Pour (self-cured), Vertex SC: Vertex Self-Cured.

**Table 2:** Mean flexural strength (MPa) and standard deviation for each experimental group.

Material	Nanoparticle concentration	Mean flexural strength	Standard deviation	*p*-value (compared to the control)
PROBASE	Control	102.4	±9.2	–
	0.3%	107.3	±8.5	0.32
	0.5%	111.7	±10.4	0.01
	1%	114	±10.2	0.008
DPI HC	Control	91.7	±2.6	–
	0.3%	93.2	±2.8	0.07
	0.5%	96.4	±4.7	0.007
	1%	98.5	±6.7	0.0026
VERTEX HC	Control	92.1	±1.8	–
	0.3%	95.6	±2.6	0.36
	0.5%	99.5	±2.5	0.02
	1%	102.8	±4.1	0.003
GC SC	Control	70.4	±1.6	–
	0.3%	73.5	±3.3	0.77
	0.5%	76.1	±1.9	0.04
	1%	77.5	±1.8	0.000
VERTEX SC	Control	69.5	±3.5	–
	0.3%	72.3	±2.8	0.22
	0.5%	75.3	±4.4	0.008
	1%	78.6	±4.3	0.002

In contrast, incorporation of 0.5% zMgO NPs resulted in statistically significant increases in flexural strength compared to controls across all acrylic resin types (*p* < 0.05 for all groups; Probase HC: *p* = 0.01; DPI HC: *p* = 0.007; Vertex HC: *p* = 0.02; GC SC: *p* = 0.04; Vertex SC: *p* = 0.008). Similarly, the 1% zMgO NPs group had a statistically significant increase in flexural strength compared to the controls across all acrylic resin types (*p* < 0.05 for all groups; Probase HC: *p* = 0.008; DPI HC: *p* = 0.0026; Vertex HC: *p* = 0.003; GC SC: *p* = 0.000; Vertex SC: *p* = 0.002).

Pairwise comparisons between nanoparticle concentrations further confirmed this trend. Samples containing 0.5% and 1% zMgO NPs demonstrated significantly higher flexural strength than those containing 0.3% across all materials (*p* < 0.05). Additionally, 1% zMgO NPs produced significantly higher flexural strength compared to 0.5% in all groups (*p* < 0.05), indicating a progressive, concentration-dependent reinforcement effect.

## Discussion

Edentulism continues to represent a prevalent clinical condition, particularly in aging populations, and prosthetic rehabilitation frequently relies on acrylic resin–based dentures. Although PMMA remains the standard denture base material, mechanical failure is a persistent clinical concern. Fracture events are multifactorial and can result from cyclic masticatory loading, structural design limitations, material fatigue, and accidental trauma (46). Previous reinforcement strategies have attempted to improve mechanical behavior through chemical modification of the polymer matrix, including crosslinking or rubber copolymerization. While such approaches may enhance impact resistance, they may also adversely affect other mechanical parameters such as flexural strength and fatigue resistance, highlighting the difficulty of optimizing multiple properties simultaneously (20, 47).

The inclusion of nanoparticles in denture bases has been increasingly studied over the last decade. These nanoparticles can either be incorporated into denture base resin during mixing or utilized as coatings on the intaglio surface of the prosthesis to minimize microbial attachment and biofilm development (7, 48). Depending on formulation characteristics, the fillers may restrict polymer mobility, redistribute applied stress, and improve resistance to deformation (20, 26, 49). For metal oxide nanoparticles, both the concentration and size must be adequately low to ensure homogeneous mixtures (50, 51). Uniform dispersion is especially important because aggregation can produce stress concentration sites that compromise structural integrity (52).

In the present study, low concentrations of zMgO NPs were selected because these levels (0%, 0.3%, 0.5%, and 1%) previously demonstrated antimicrobial activity while maintaining material stability (35). Earlier investigations using similar nanoparticle systems reported significant reductions in microbial adhesion without evidence of particle leaching, suggesting stable incorporation within the polymer matrix (37). Additionally, we previously found that Scanning Electron Microscopy revealed that surface coatings helped achieve more uniform nanoparticle dispersion and reduce agglomeration, thereby improving interfacial bonding between the filler and the polymer matrix. Moreover, Fourier Transform Infrared Spectroscopy analyses detected changes in peak intensity patterns after nanoparticle incorporation, indicating interaction between inorganic fillers and resin components (34, 37, 53).

It is important to highlight that heat-cured denture base materials are subjected to high heat and pressure, which leads to extended polymer chains, lower levels of residual monomer, and better degrees of monomer conversion. In contrast, autopolymerizing denture base materials typically contain excess monomers that can interact with the inorganic nanoparticles, leading to increased peak heights and variations in peak intensity (5, 7).

Meanwhile, zein is a completely amorphous polymer that exhibits plasticizing viscoelasticity and a glass transition temperature of about 165 °C, although this glass transition temperature decreases significantly as the degree of plasticization increases. Its degradation point is 270 °C, which is much higher than the polymerization temperature of the acrylic resin, which should never exceed 100 °C to avoid monomer evaporation at 100.3 °C (54–56).

Flexural forces occur during the everyday use of dentures, as they are subjected to repeated bending, flexing, and twisting. These forces can increase in the presence of different bone resorption patterns, creating an uneven landscape among the alveolar ridges. Therefore, denture bases must possess adequate flexural strength to extend their clinical viability (57). This may further be influenced by the chemical composition of the acrylic resin, the curing method, the degree of polymerization, and storage conditions (6, 7).

Regardless of material type, all nanoparticle-reinforced sample groups demonstrated increased flexural strength compared to the control, with the 0.5% and 1% groups exhibiting a statistically significant increase in strength compared to the unmodified acrylic groups. Therefore, the null hypothesis was rejected. The minimum flexural strength value of standard dental base polymers is mandated to be 65 MPa by the International Organization for Standardization ISO-20795-1 (9, 45, 58). All groups, both modified and unmodified, met this threshold. This pattern suggests a threshold effect in which reinforcement becomes measurable only once sufficient nanoparticle content is present to influence internal stress distribution. When nanoparticles are evenly distributed, they can act as barriers to crack propagation and reduce localized strain accumulation. At lower concentrations, particle spacing may be insufficient to produce measurable reinforcement, which could explain why statistically significant improvements were only detected at higher loading levels.

In this study, both heat-cured and self-cured acrylic resins were used to compare their effects. Heat-polymerized acrylic resins exhibited consistently higher flexural strength values compared to the autopolymerizing resins across all nanoparticle concentrations. The magnitude of improvement was more pronounced in heat-cured materials. Self-cured resins presented lower baseline values and smaller absolute gains, although a similar nanoparticle concentration-dependent trend was observed. This could be due to the inherent polymerization efficiency of heat-cured and self-cured acrylic resins. Heat-polymerized resins typically exhibit lower residual monomer content and a denser polymer network, which can contribute to improved mechanical performance and may enhance interfacial interactions with reinforcing agents such as nanoparticles (59, 60). Conversely, self-cured acrylic resins tend to exhibit higher porosity and residual monomer content due to their chemical polymerization mechanism, which can negatively affect their structural integrity and limit reinforcement efficiency (61, 62).

The findings highlight the importance of interfacial compatibility between nanofillers and polymer matrices. Reinforcement is not solely dependent on nanoparticle presence but on the efficiency of load transfer across the filler-matrix interface. Surface modification strategies that improve bonding may therefore be as critical as nanoparticle concentration in determining final mechanical performance. Moreover, from a clinical perspective, improved flexural strength may translate into enhanced resistance to midline fracture. Materials that maintain mechanical integrity under cyclic loading may reduce repair frequency, clinical chair time, and long-term costs for patients. Therefore, nanoparticle-modified denture bases could provide practical advantages beyond laboratory performance metrics.

The literature indicates that varying types and proportions of nanoparticles may enhance or diminish the flexural strength of acrylic resins. Ansarifard et al. reported a statistically significant increase in flexural strength when including 50 and 500 μg/mL copper oxide NPs in PMMA (63). Karci et al. investigated the change in flexural strength of different acrylic resins when adding Al_2_O_3_, SiO_2_, and TiO_2_ nanoparticles. There was an increase in flexural strength at 1% with the Al_2_O_3_ and TiO_2_ groups surpassing the SiO_2_ group, but a decrease at 3% and 5% with all nanoparticles (52). Alzayyat et al. incorporated 0.05%, 0.25%, 0.5%, and 1% SiO_2_ NPs with acrylic powder, and found that the inclusion of the nanoparticles significantly increased the flexural strength, although the degree of increase significantly reduced with higher concentrations (23).

Similarly, Gad et al. prepared 0.25%, 0.5%, and 0.75% SiO_2_ NPs with acrylic denture bases and found a significant increase in the flexural strength of all groups (24). In another study, Gad investigated the effects of adding 0.5%, 1%, and 1.5% ZrO_2_ NPs and Ag NPs to heat-polymerized acrylic resin samples. The flexural strength significantly increased proportionally to the concentration of ZrO_2_ NPs, whereas the opposite effect was seen with the Ag NPs, with the flexural strength decreasing proportionally to the concentration of Ag NPs (21). Palaskar et al. assessed 0.1%, 0.2%, 0.3%, 0.4%, and 0.5% Ag NPs with heat-cured PMMA and found that flexural strength decreased as the percentage of Ag NPs increased, with the control group having the highest flexural strength (64). Albasarah et al. evaluated the effect of adding 2.5% and 5% ZrO_2_ NPs to PMMA denture bases on flexural strength at different material thicknesses. They concluded that the addition of the nanoparticles significantly improved flexural strength, even with reduced material thickness (65). Such discrepancies likely reflect differences in nanoparticle chemistry, concentration ranges, processing methods, and dispersion quality. These findings underscore the importance of optimizing formulation parameters rather than assuming uniform reinforcement effects across nanoparticle types.

Despite the promising attributes of nanoparticle-enhanced denture materials, several limitations should be considered. This study was conducted under *in vitro* conditions and did not fully replicate the complex intraoral environment, where denture base materials are subjected to thermal fluctuations, moisture, enzymatic activity, and cyclic masticatory loading. In addition, the present investigation focused solely on flexural strength, which, although clinically relevant, does not comprehensively represent the mechanical performance of denture base materials.

Future studies should include simulated oral conditions, including thermocycling, mechanical fatigue loading, and exposure to saliva, to better approximate clinical performance. Long-term investigations evaluating multiple mechanical and physical properties, such as impact strength, fracture toughness, fatigue resistance, and wear behavior, could reveal much on the durability of the nanoparticle-polymer interface. These future investigations are necessary to determine the translational potential and clinical applicability of these nanoparticle-modified denture materials.

## Conclusion

This study evaluated the effect of zMgO NPs on the flexural strength of PMMA denture bases to address the need for materials with improved mechanical and antimicrobial properties. The results demonstrated that incorporation of 0.5% and 1% zMgO NPs significantly increased flexural strength across multiple PMMA types. No significant improvement was observed at 0.3%, indicating a concentration-dependent effect. While these findings suggest that zMgO NPs could improve flexural strength, further research is needed to assess additional properties such as impact strength, fracture toughness, fatigue resistance, and long-term aging under simulated oral conditions to determine the overall performance and clinical relevance of these materials.

## Data Availability

The original contributions presented in the study are included in the article/Supplementary Material, further inquiries can be directed to the corresponding author.
